# Influence of Climatic Factors on Malaria Epidemic in Gulu District, Northern Uganda: A 10-Year Retrospective Study

**DOI:** 10.1155/2018/5482136

**Published:** 2018-08-13

**Authors:** Ouma Simple, Arnold Mindra, Gerald Obai, Emilio Ovuga, Emmanuel Igwaro Odongo-Aginya

**Affiliations:** ^1^School of Public Health, College of Health Sciences, Makerere University (MUK), P.O. Box 7072, Kampala, Uganda; ^2^Mbarara University Research Training Initiative, Mbarara University of Science and Technology (MUST), P.O. Box 1410, Mbarara, Uganda; ^3^Department of Physiology, Faculty of Medicine, Gulu University, P.O. Box 166, Gulu, Uganda; ^4^Department of Psychiatry, Faculty of Medicine, Gulu University, P.O. Box 166, Gulu, Uganda; ^5^Department of Immunology and Microbiology, Faculty of Medicine, Gulu University, P.O. Box 166, Gulu, Uganda

## Abstract

**Background:**

Globally, 15 countries, mainly in Sub-Saharan Africa, account for 80% of malaria cases and 78% of malaria related deaths. In Uganda, malaria is endemic and the mortality and morbidity due to malaria cause significant negative impact on the economy. In Gulu district, malaria is the leading killer disease among children <5 years. In 2015, the high intensity of malaria infection in Northern Uganda revealed a possible link between malaria and rainfall. However, available information on the influence of climatic factors on malaria are scarce, conflicting, and highly contextualized and therefore one cannot reference such information to malaria control policy in Northern Uganda, thus the need for this study.

**Methods and Results:**

During the 10 year's retrospective study period a total of 2,304,537 people suffered from malaria in Gulu district. Malaria infection was generally stable with biannual peaks during the months of June-July and September-October but showed a declining trend after introduction of indoor residual spraying. Analysis of the departure of mean monthly malaria cases from the long-term mean monthly malaria cases revealed biannual seasonal outbreaks before and during the first year of introduction of indoor residual spraying. However, there were two major malaria epidemics in 2015 following discontinuation of indoor residual spraying in the late 2014. Children <5 years of age were disproportionally affected by malaria and accounted for 47.6% of the total malaria cases. Both rainfall (P=0.04) and relative humidity (P=0.003) had significant positive correlations with malaria. Meanwhile, maximum temperature had significant negative correlation with malaria (P=0.02) but minimum temperature had no correlation with malaria (P=0.29).

**Conclusion:**

Malaria in Gulu disproportionately affects children under 5 years and shows seasonality with a generally stable trend influenced by rainfall and relative humidity. However, indoor residual spraying is a very promising method to achieve a sustained malaria control in this population.

## 1. Background

Malaria is an acute public health problem in many countries. Globally, about half of the world's populations are at risk of malaria infection [1]. Sub-Saharan Africa carries high global malaria burden. Children living in these poorest places die of malaria related deaths due to lack of access to diagnostic test and quality treatment [2]. However, Insecticide Treated Nets (ITNs) accounted for an estimated 68% of malaria cases prevented across Africa since 2000. However, 25% of children in Sub-Saharan Africa still live in households without ITN [3]. Another method of malaria infection control is use of indoor residual spraying (IRS) which is a very effective method, especially, in high-transmission areas [4]. Among children <5 years of age in Uganda, IRS was associated with reduction in both malaria parasitaemia and anaemia [5].

Uganda has one of the highest malaria transmission rates in the world and is third in malaria mortality in Africa [6]. Among outpatient department (OPD) attendees in 2009, 41% of Ugandans were tested positive for malaria parasite [7]. During the same period, there was high rate of consumption of antimalaria drugs leading to stock-outs of most antimalarial drug and high stock-out rates of quinine (15%), Fansidar (13%), and the first-line antimalaria drugs (13%) {Artemether+Lumefantrine and Artesunate+Amodiaquine}. Likewise, in 2009/2010, malaria morbidity among Ugandans was put at 48%, where more than 13million people were infected with malaria [7], an increase from 38.7% in 2002 [8]. The high morbidity of malaria in Uganda causes significant negative impact on economy and puts heavy economic burden on individuals and households [9]. According to the Ministry of Health (MoH), a significant percentage of unreported deaths due to malaria occurs at home and 27.2% of inpatient deaths among children <5 years are due to malaria especially in Northern Uganda which had the highest malaria prevalence of 63% among children <5 years [10]. In 2013, malaria was the leading killer disease (27%) among children <5 years of age in Gulu district and at the same time the greatest cause of morbidity of 34% in the general population with 86 functional health facilities serving 443,733 people [11].

Temperature and rainfall are two climatic factors previously used to forecast malaria outbreaks in East Africa [12, 13]. Gomez-elipe et al. (2006) found that, in Burundi, both rainfall and maximum temperature were positive predictors of malaria whereas minimum temperature had no effect on malaria [14]. Another study in Burundi highlands, however, showed that higher minimum temperature actually resulted in higher prevalence of* Anopheles *mosquitoes responsible for malaria transmissions [15]. In Zimbabwe, both rainfall and relative humidity were found to be positive predictors of malaria infection, while maximum temperature and minimum temperature were both negative predictors of malaria infection [16].

In Delhi, mean rainfall and relative humidity, both lagged at 1 month, were significant predictors of malaria infections but maximum temperature did not significantly affect malaria infections [17]. Malaria infection is affected by vector load which is highly dependent on relative humidity and rainfall [18, 19]. Moreover, the influence of climatic factors on malaria infection may differ from place to place based on many local contexts like availability of good public health system for malaria prevention and treatment, socioeconomic factors, and local land use [15, 20]. These conflicting findings on the influence of climatic variables on malaria infection, therefore, make it difficult for one to reference the different literature to malaria control policy in Northern Uganda without consideration to the local contexts.

## 2. Materials and Method

### 2.1. Description of the Study Area

**Figure 1:**
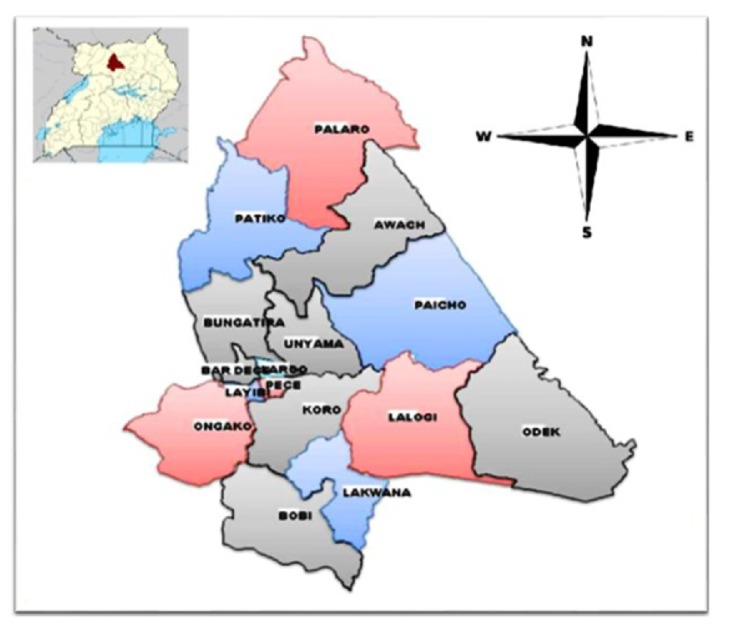
Showing the different subcounties in the study district of Gulu. Map of Uganda showing Gulu district is in the Left upper corner.

This study was carried out in Gulu district, Northern Uganda. Gulu district is comprised of three counties of Gulu Municipality, Aswa County, and Omoro County. The total population in the district stood at 443,733 out of whom 228,123 (51.4%) were females [11] (Figure 1).

Gulu district is located between longitudes 30-32 degrees East and latitudes 2-4 degrees North. The altitude ranges between 1000 and 1200 meters above sea level. The type of climate experienced in Gulu consists of alternate dry and wet seasons. The wet seasons extend from April to November, while the dry seasons extend from November to March. The vegetation is intermediate savannah grassland and more than 80% of the population in the district practice subsistence farming [21].

### 2.2. Study Design

In this retrospective study, the monthly records of malaria cases and mean monthly climatic variables (maximum temperature, minimum temperature, rainfall, and relative humidity) over the previous 10 years period between January 2006 and December 2015 were utilized.

### 2.3. Ethical Review

Gulu University Research Ethics Committee (GUREC) waived the study from ethical review since it did not directly involve human subjects. Gulu district biostatistician in the District Health Office and the officer in charge of Uganda National Metrological Authority (UNMA) gave permission to extract data on malaria and climatic variables, respectively.

### 2.4. Description of the Study Materials and Methods

Monthly cases of malaria from January 2006 to December 2015 were extracted from the Health Management Information System (HMIS) at Gulu district biostatistics office. Data were categorized as malaria in pregnancy, malaria cases among male children <5 years, malaria cases among female children <5 years, malaria cases in the rest of male population ≥5 years, and malaria cases in the rest of female population ≥5 years. Likewise, UNMA provided the 10-year retrospective mean monthly rainfall, mean monthly relative humidity, mean monthly maximum temperature, and mean monthly minimum temperature for the corresponding months. Sorted data was entered into SPSS 16.0.

### 2.5. Data Analysis

Both univariate and bivariate analyses were carried out using SPSS 16.0. Frequencies and proportion were calculated for malaria cases and climatic variables. Sequence charts were created to show the trends in malaria morbidity, rainfall, and relative humidity. In the bivariate analysis, Pearson correlation coefficient tests were carried out to determine correlation among climatic variables and their individual influence on malaria. A P value of <0.05 was considered statistically significant. The departure from the long-term (10 years) mean monthly malaria cases was calculated by subtracting the long-term (10 years) mean monthly malaria cases from individual mean monthly malaria cases and a departure of <0% was considered effective malaria control, a departure of >0% but <100% was considered an outbreak, and a departure of ≥100% was considered an epidemic. The annual population in Gulu district as used in calculations was estimates from the 2013 census data showing population of 443,733 in the district and annual population growth rate of 3.2% during the study period. Results are presented as frequencies, proportions, and graphs.

## 3. Results

### 3.1. Malaria and Indoor Residual Sparing

**Figure 2:**
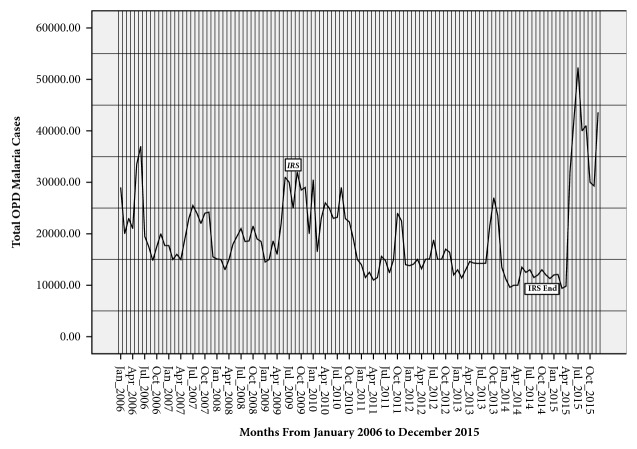
Showing malaria time series with interposed IRS program in Gulu district.

The total outpatient department (OPD) malaria cases reported during the 10-year study period in Gulu stood at 2,304,537 cases with a 10-year (long-term) mean monthly malaria cases of 19,205. Therefore, in a district of an estimated 410,725 people, monthly, malaria affected more than 4.7% (19,205/410,725) of the total population. Thus, cumulatively, more than half of the population (56%) suffer from malaria each year. Moreover, sequence charts showed that malaria was endemic in the district but generally stable with biannual peaks in the months of June-July and October-November (Figures 2 and 5).

Before introduction of IRS, there were numerous (<100%) biannual proportional departures of mean monthly malaria cases from the long-term mean monthly malaria cases. These show that there were many seasonal malaria outbreaks in the district. However, from 2009, IRS effectively reduced the annual proportion of population of Gulu district affected by malaria from 71.5% (278,664/ 389,604) in 2009 to 29% (133,000/457,934) in 2014. However, two serious malaria epidemics were recorded in 2015 where 74.1% (350,000/472,586) of entire population of Gulu suffered from malaria. This was a big and sudden increase in the annual proportion of population affected by malaria from the 2014 level when IRS, for malaria prevention, was being used. The two malaria epidemics were revealed by the two instances of more than 100% increase in the proportional departures from the long-term mean monthly malaria cases during the months of June to September 2015 and December 2015. The first malaria epidemic started in June 2015, 9 months after the discontinuation of IRS in September 2014 (Figure 5).

**Figure 3:**
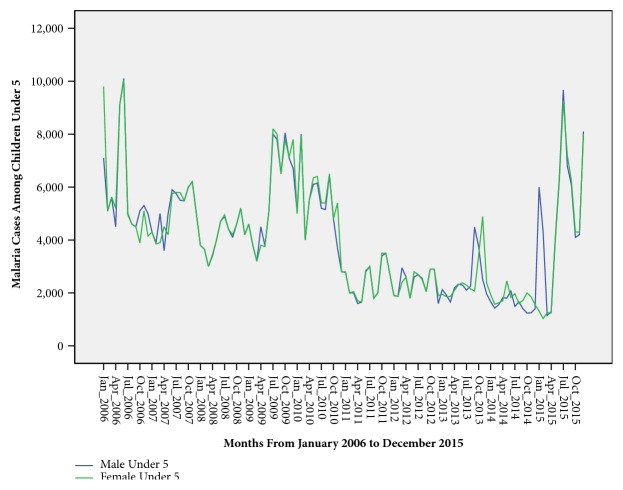
Time series showing variation of malaria burden among children under 5 years of age.

**Figure 4:**
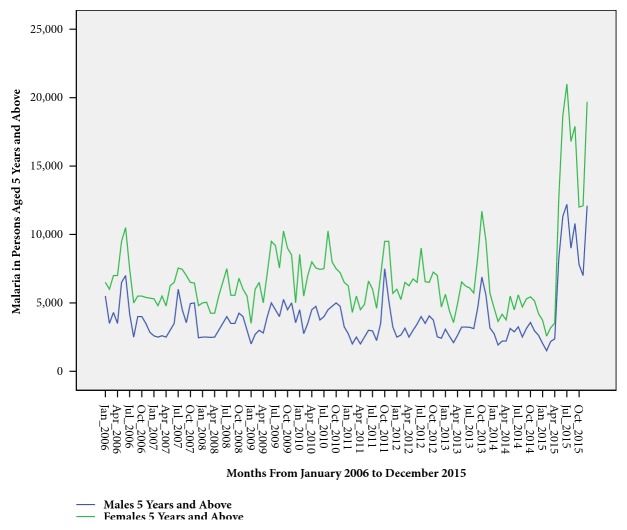
Time series showing malaria burden among persons 5 years and above.

Children <5 years accounted for 47.6% (968,882/2034537) of the total malaria cases reported in the district. There was no sex predisposition to malaria infection among children <5 years of age (Figure 3). However, unlike children <5 years of age, females ≥5 years of age were twice more affected by malaria than their male counterpart (Figure 4).

### 3.2. Climatic Factors and Malaria

The average mean monthly rainfall in Gulu was 110 mm with a range from zero in January 2015 to 381 mm in August 2013. Gulu exhibited two seasons during study period: a longer wet season spanning from March to November and a shorter dry season spanning from December to February. The rainy season has two peaks; smaller peaks of average mean monthly rainfall of 150 mm April/May and heavier peak of average mean monthly rainfall of 234 mm in August/September. Rainfall showed a significant positive correlation with relative humidity (P<0.001) and the mean minimum temperature showed a significant positive correlation with mean maximum temperature (P<0.01). Both mean maximum temperature and mean minimum temperature showed a significant negative correlation with rainfall (P<0.001). However, only mean maximum temperature showed a significant negative correlation with relative humidity (P<0.001) unlike the mean minimum temperature which showed no significant correlation with relative humidity.

**Figure 5:**
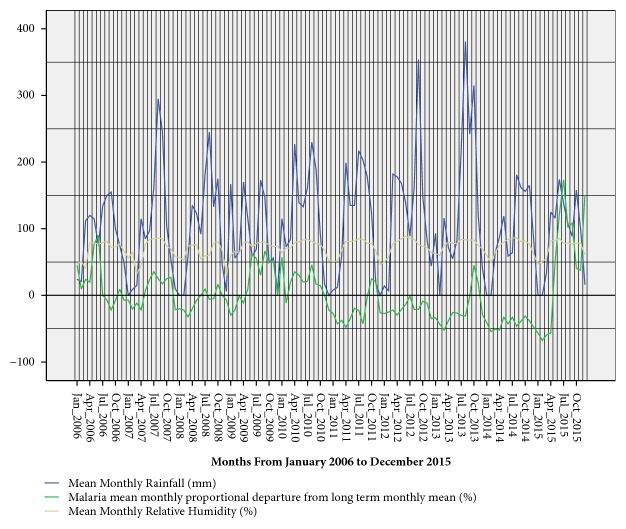
Showing relationship of long-term (10 years) malaria mean monthly cases with rainfall and relative humidity.

Among all study groups, there were seasonal variations of malaria cases with a generally decreasing trend. Critical observation of trends of malaria outbreaks and epidemics with respect to rainfall amount indicates that mean monthly rainfall amount of at least above the 10-year mean monthly level of 110 mm is required for either an outbreak or an epidemic to occur. Nevertheless, the influence of rainfall on malaria infection differed by age group and pregnancy status. Rainfall showed significant positive correlation with malaria in pregnancy (P=0.04) and malaria among person ≥5 years of age (P=0.04). However, there was no significant correlation between rainfall and malaria in children <5 years of age. Rainfall peaks lagged behind malaria peaks by 1-2 months. Amount of rainfall greatly affects the magnitude of malaria infection whereby the first seasonal malaria outbreaks in June/July, just 1-2 months after the firsts and smaller rainfall peak, had higher malaria infection than the second seasonal malaria outbreaks in October/November, 1-2 months after the second and higher rainfall peak. However, introduction of IRS resulted in reversal in the magnitudes of the seasonal malaria peaks with the first malaria outbreaks having lower magnitude than the second outbreaks (Figure 5).

There was high relative humidity from April to November which coincided with rainy season and low relative humidity between December and March which coincided with the dry season. The average mean monthly relative humidity during the study period was 71.8% (Figure 5). Relative humidity showed significant positive correlation with malaria (P=0.003). However, unlike in the rest of the population, there was no correlation between relative humidity and malaria in children <5 years.

The average mean monthly maximum temperature was 30.4°C while the average mean minimum temperature was 19.1°C. Mean monthly maximum temperature was statistically significant negative predictor of malaria in the general population (P=0.02), malaria in pregnancy (P=0.004), and malaria among persons ≥5 years (P=0.01). There was no statistically significant correlation between mean maximum temperature and malaria infection among children <5 years. Mean monthly minimum temperature showed no significant correlation with malaria in all the study categories.

## 4. Discussion

During the 10-year study period, malaria cases were reported each and every month throughout the year but with biannual seasonal outbreaks and two major consecutive malaria epidemics just 9 months after discontinuation of IRS. Children <5 years of age were disproportionally affected by malaria, accounting for 47.6% of population who had malaria but with no sex predilection. However, females 5 years and above of age suffered from malaria twice more than their male counterparts. Rainfall and relative humidity showed significant positive correlations with malaria, whereas mean maximum temperature showed significant negative correlation with malaria unlike the mean minimum temperature which showed no correlation with malaria.

The study revealed that malaria infection is endemic in Gulu district. This result is in line with previous report showing that 90- 95% of Uganda, including Gulu district, have stable perennial malaria transmission [6] probably due to the favourable local relative humidity for mosquitoes' survival as seen in Gulu with a mean monthly relative humidity of 71.8% which is well above the minimal conducive relative humidity (60%) best for mosquitoes' survival [21, 22]. Moreover, this study also indicated that both mean monthly rainfall and relative humidity were significant positive drivers of malaria in Gulu district. The effect of rainfall on malaria is probably through its intimate relationship with the relative humidity.

Children <5 years of age were disproportionately affected by malaria. This could be attributed to declining maternal immunity to malaria infection especially after age of 2 years as evidenced in other previous studies [21–23]. Malaria in children <5 years of age showed no sex predilection just like with a Nigerian study where there was no significant difference in malaria infections among male and female children [24]. However, compared to children <5 years of age, females of 5 years and above were twice more likely than their male counterparts to suffer of malaria. The higher cases of malaria are attributable to gender roles in Acholi culture as predominantly practiced in Gulu district. In Acholi culture, just like in most African cultures, women are the ones who predominantly do the household chores, staying up deep into the night and waking up very early in the morning, thus getting more exposed to the early morning mosquito bites.

Indoor residual spraying resulted in reduction in malaria cases by more than half (52.3%) during the 5 years of its utilization to control malaria. The greatest reductions in malaria infections were during the high-transmission first seasonal malaria peaks. This resulted in relatively more cases of malaria recorded in the second seasons of malaria outbreak compared to pre-IRS when more malaria cases were reported during the first seasonal malaria outbreak. This finding is in agreement with other studies showing that effect of IRS is more marked in high-transmission settings [7, 25]. However, the current finding revealing reversal of malaria seasonality, from highest malaria peaks being in the first rainy seasons before IRS introduction to highest malaria peaks being in second rainy season with introduction of IRS, is rather an unreported phenomenon. However, the dramatic change in malaria cases demonstrated that there is unquestionable need to sustain IRS as evidenced in Southern Africa in order to achieve malaria eradication [26].

Moreover, this study suggests that even with good coverage of LLINs of 89%, well above the Presidential Malaria Initiative (PMI) target 85% LLINs coverage in Uganda [27, 28], LLINs alone was not effective in preventing malaria resurgence. This is because of the favourable local weather conditions for both vectors and parasites reproduction and survival, which increases the risk of infected mosquito bite. Furthermore, without the use of second intervention of either seasonal malaria chemoprophylaxis or IRS, LLINs alone is found not to be sufficient in malaria reduction in high-transmission settings [29]. A study in Burundi showed that high* Anopheles* density, due to absence of vector control methods, is a single most important predictor of malaria infections [15]. In addition, using both LLINs and IRS offers greater protection than either LLIN or IRS alone [26, 27]. Therefore, in a situation where choice has to be made between IRS and LLINs, IRS is found to be both more effective and efficient than LLINs [30].

In this study, both rainfall and relative humidity showed significant positive correlations with malaria infection. Both Nwaorgu O.C et al. (2011) and Ngomane N et al. (2012) found significant positive correlation of malaria infection with both rainfall and relative humidity [24, 31]. Rainfall significantly increases the number of infective* Anopheles* mosquitoes with P.* falciparum* malaria parasites [32]. Rainfall peaks were found to lag behind malaria peaks by 1-2 months. Just like in South India, where Kumar DS et al. (2014) found that rainfall lagged behind malaria by 1-2 months, many other studies came out with the similar results [20, 25, 27, 28, 30]. However, the higher rainfall amount resulted in lower malaria infection whereas lower rainfall amount resulted in higher malaria infection. This is because lower rainfall is conducive for occurrence of higher* Anopheles *density [15] as opposed to higher rainfall which tends to flush and kill the A*nopheles *larvae out of their aquatic habitats resulting in lower* Anopheles* density and thus reduced rate of malaria transmission [33].

Mean maximum temperature showed negative correlation with malaria just like in Bangladesh and Iran where increase in mean maximum temperature was associated with decline in* Anopheles *mosquito and malaria infection [34, 35]. Likewise, in Bhutan mean maximum temperature was a good predictor of malaria infection [23]. Average mean maximum temperature in Gulu was 30.4°C, well above the conducive temperature for largest abundance of long-lived mosquitoes which is across the 20-30°C temperature range and drops at higher temperature [36]. Increasing mean maximum temperature results in excessive heat, which reduces malaria reproduction and survival. The reduction in the number of malaria parasites, due to poor reproduction and survival, lowers the potential for malaria transmission in case of infective mosquito bites. Thus, there is reduction in malaria transmission [36]. Just like in this current study, mean minimum temperature did not predict malaria infections in Nigeria [37]. However, in Zimbabwe, minimum temperature was negative predictor of malaria infection [16].

## 5. Strengths and Limitations

The strength of the study is that data was collected over a long period of time from a large sample size. Thus, the information generated is representative of the actual malaria situation on the ground. However, by analysing secondary data, the authors may not guarantee accuracy of the information used since data captured in the registry might be inaccurate, incomplete, and poorly entered. Secondly, many malaria cases could have been managed either at home or in other health facilities where Health Management Information System did not capture malaria cases.

## 6. Conclusions

Malaria in Gulu district showed seasonal variations predictable by rainfall and relative humidity and that children under 5 years of age bear the greatest burden of malaria. However, there is need for further studies to develop malaria early warning system based on the above climatic variables. These will provide information on good malaria prevention policy as well as good malaria case management planning. Secondly, in absence of seasonal malaria chemoprevention in malaria high-transmission settings like Gulu, IRS should be the secondary malaria preventive method alongside LLINs in order to attain significant reduction in malaria morbidity.

## Data Availability

The data used to support the findings of this study are available from the corresponding author upon request.
